# Water fleas stand the heat and stay in the city

**DOI:** 10.1093/conphys/cox059

**Published:** 2017-10-11

**Authors:** Natalie Sopinka

**Affiliations:** 1 Canadian Science Publishing, 65 Auriga Dr, Suite 203, Ottawa, ON, Canada K2E 7W6

One of the most dramatic visuals of the 21st century is the sprawling skyline of a city. Glass-paned buildings as you look up, endless pavement as you look down. Cars honk. Tailpipes leak out pollutants. Lightbulbs buzz as a metropolis works into the night. What you do not necessarily see or hear… but might feel… is the heat. Because of human activities, there is an increase in air temperatures as you move into a city from its rural edges. This urban-rural temperature gradient is called the ‘urban heat island’ effect.

With urbanization and climatic warming becoming the new reality, Brans *et al.* wanted to find out how well urban animals cope with the heat. The team used water fleas in ponds surrounding the city of Brussels, Belgium as their model. Can urban fleas handle the heat? Yes!

But, measuring the heat tolerance of water fleas was no easy task, requiring tremendous patience and thousands of sample vials.

The researchers collected fleas from 12 different ponds—some near the heart of the city in built-up urban areas, others more distant, in less built-up rural areas. They cultured the fleas in the laboratory in either 20°C or 24°C water and determined their heat tolerance. To do this, they placed individual fleas from each pond population and water temperature into vials, heated the vials, and observed the temperature at which the flea stopped swimming and sank.

Fleas from urban ponds had nearly 2°C higher heat tolerance than the fleas from rural ponds. This finding suggests that some fleas have adapted to the ‘urban heat island’ effect, tolerating the warmer temperatures of city life. When both urban and rural fleas were raised at 24°C, they both had higher heat tolerance than if either population was raised at 20°C. This shows that thermal tolerance can be flexible within one generation.

What is the underlying mechanism responsible for this temperature adaptation in fleas? As aquatic environments get warmer, oxygen supply decreases. So, Brans *et al.* thought that the fleas living in warmer waters would have to enhance their ability to acquire and transport oxygen. When compared with rural fleas, urban fleas did have a higher concentration of haemoglobin, the key oxygen-transporting protein that we humans also have in our blood. But, even though the fleas raised at 20°C and 24°C showed different thermal tolerance, they did not have different haemoglobin concentrations. The researchers think there are likely other proteins that help fleas tolerate warmer temperatures.

Fleas may be little, but their role in urban pond food webs is mighty—they are bite-sized snacks for baby fishes, which are bite-sized snacks for larger fishes and birds. Also, as ferocious grazers of algae, fleas contribute to urban beautification, keeping urban ponds clear.

The full mechanism behind the flea’s thermal tolerance may not be known yet, but if fleas can handle the heat, perhaps the ecosystem services they provide can persist as the skylines continue to sprawl.

**Figure:**
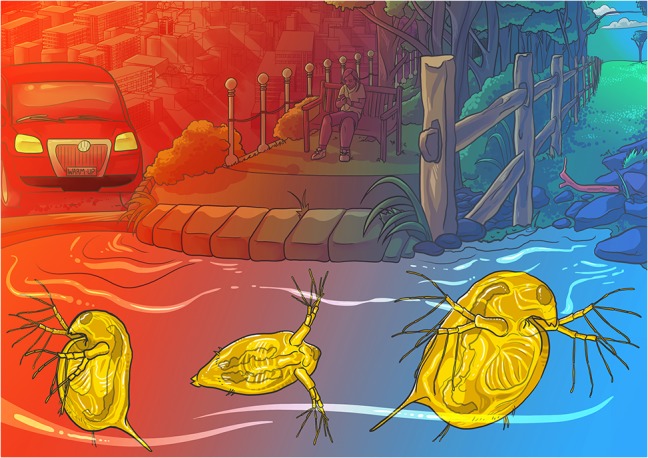


Illustration by Erin Walsh; Email: ewalsh.sci@gmail.com
